# Supplementary material for: Socio-economic condition and lack of virological suppression among adults and adolescents receiving antiretroviral therapy in Ethiopia
Source: PLoS One. 2020 Dec 15;15(12):e0244066. doi: 10.1371/journal.pone.0244066 (PMC7737988; doi:10.1371/journal.pone.0244066)
Supplement: S1 Table — (DOCX) [file pone.0244066.s001.docx]

**S1 Table. Comparison of participants who could be reached and included, with eligible participants who could not be reached (unavailable medical record or not reached by phone or home visit) in terms of demographic and medical characteristics, divided by viral load category.**

|  | Eligible cases, not reached  (n=38) | p-value^¶^  (*compared to cases reached*) | Eligible controls, not reached  (n=55) | p-value^¶^  (*compared to controls reached*) |
| --- | --- | --- | --- | --- |
| Age (years; median, IQR) | 38 (33-44) | 0.743 | 43 (33-50) | 0.401 |
| Gender |  | 0.725 |  | 0.805 |
| Male | 16 (43.2) |  | 18 (32.7) |  |
| Female | 21 (56.8) |  | 37 (67.3) |  |
| Pre-ART CD4 cell count^†^ (cells/µl; median, IQR) | 221 (123-470) | **0.006** | 310 (176-442) | 0.108 |
| Recent CD4 cell count, <1 year^‡^ (cells/µl; median, IQR) | 289 (128-624) | 0.584 | 492 (360-710) | 0.915 |
| Duration of ART (years; median, IQR) | 10.1 (6.4-11.7) | 0.153 | 6.1 (2.6-10.3) | 0.118 |
| ART regimen |  | 0.477 |  | 0.969 |
| First-line | 28 (93.3) |  | 52 (98.1) |  |
| Second-line regimen | 2 (6.7) |  | 1 (1.9) |  |
| Viral load (copies/ml; median, IQR) | 6402 (1786-37592) | 0.673 | <150 |  |
| 1000-10,000 | 22 (61.1) |  |  |  |
| 10,000-100,000 | 11 (30.6) |  |  |  |
| >100,000 | 3 (8.3) |  |  |  |

Binominal logistic regression for categorical variables. Mann-Whitney U test for continuous variables. Data reported in absolute number and percentage (in brackets) and unadjusted odds ratios, unless otherwise specified. CD4: CD4 cell count; IQR: Interquartile range; ART: Antiretroviral therapy; NNRTI: Non-Nucleoside Reverse Transcriptase Inhibitor.

† Missing values of pre-ART CD4: 2 (uncontacted cases); 6 (uncontacted controls)

‡ Missing values of recent CD4 (within 1 year): 31 (uncontacted cases); 46 (uncontacted controls)

¶ Data from cases and controls available in table 1.
