# Supplementary material for: Socio-economic condition and lack of virological suppression among adults and adolescents receiving antiretroviral therapy in Ethiopia
Source: PLoS One. 2020 Dec 15;15(12):e0244066. doi: 10.1371/journal.pone.0244066 (PMC7737988; doi:10.1371/journal.pone.0244066)
Supplement: S2 Table — (DOCX) [file pone.0244066.s002.docx]

**S2 Table.** **Comparison of demographic and medical characteristics of included study participants by viral load category and gender.**

|  | Male | | Female | | p-value (males) | p-value (females) |  |
| --- | --- | --- | --- | --- | --- | --- | --- |
|  | **Cases (72)** | **Controls (47)** | **Cases (83)** | **Controls (105)** |  |  |  |
| Age (years; median, IQR) | 38.0 (30-45) | 47.0 (40-54) | 35.0 (30-42) | 36.0 (31-43,5) | **<0.001** | 0.277 |  |
| Gender |  |  |  |  |  |  |  |
| Male | 100 | 100 | 100 | 100 |  |  |  |
| Female | 0 | 0 | 0 | 0 |  |  |  |
| Pre-ART CD4 cell count^†^ (cells/µl; median, IQR) | 143 (83-229) | 176 (90-282) | 181 (100-316) | 246 (145-402) | 0.299 | 0.056 |  |
| Recent CD4 cell count, <1 year^‡^ (cells/µl; median, IQR) | 315 (254-454) | 459 (339-602) | 393 (238-495) | 526 (404-622) | 0.070 | **0.028** |  |
| Duration of ART (years; median, IQR) | 7.90 (5.02-11.0) | 7.94 (5.01-10.6) | 9.31 (7.22-10.8) | 7.76 (4.26-10.1) | 0.763 | **0.003** |  |
| ART regimen^§^ |  |  |  |  | 0.107 | **0.035** |  |
| First-line | 62 (86.1) | 45 (95.7) | 75 (90.4) | 104 (99.0) |  |  |  |
| Second-line regimen | 10 (13.9) | 2 (4.3) | 7 (9.6) | 1 (1.0) |  |  |  |
| Transfer-in |  |  |  |  | 0.542 | 0.137 |  |
| Yes | 12 (16.9) | 6 (12.8) | 16 (19.3) | 12 (11.4) |  |  |  |
| No | 59 (83.1) | 41 (87.2) | 67 (80.7) | 93 (88.6) |  |  |  |
| ART clinic location |  |  |  |  | 0.565 | 0.434 |  |
| Same district as residence | 55 (76.4) | 38 (80.9) | 73 (88.0) | 96 (91.4) |  |  |  |
| Different district | 17 (23.6) | 9 (19.1) | 10 (12.0) | 9 (8.6) |  |  |  |
| Viral load (copies/ml; median, IQR) | 8106 (4025-30396) |  | 5101 (1931-23022) | - |  |  |  |
| 1000-10,000 | 38 (53.5) |  | 52 (62.7) | - |  |  |  |
| 10,000-100,000 | 21 (29.6) |  | 24 (28.9) | - |  |  |  |
| >100,000 | 12 (16.9) |  | 7 (8.4) | - |  |  |  |

Binominal logistic regression for categorical variables. Mann-Whitney U test for continuous variables. Data reported in absolute number and percentage (in brackets) and unadjusted odds ratios, unless otherwise specified. CD4: CD4 cell count; IQR: Interquartile range; ART: Antiretroviral therapy; NNRTI: Non-Nucleoside Reverse Transcriptase Inhibitor.

† Missing values of pre-ART CD4: 20 (cases); 10 (controls)

‡ Missing values of recent CD4 (within 1 year): 120 (cases); 111 (controls)

§ First-line regimens (based on non-nucleoside reverse transcriptase inhibitors): Nevirapine-based: 40.9% (cases), 37.5% (controls); Efavirenz-based: 48.0% (cases), 60.5% (controls); Second-line regimens (based on protease inhibitors): Atazanavir/ritonavir-based: 9.0% (cases), 1.3% (controls); Lopinavir/ritonavir-based: 1.3% (cases), 0.7% (controls)
