# Supplementary material for: Socio-economic condition and lack of virological suppression among adults and adolescents receiving antiretroviral therapy in Ethiopia
Source: PLoS One. 2020 Dec 15;15(12):e0244066. doi: 10.1371/journal.pone.0244066 (PMC7737988; doi:10.1371/journal.pone.0244066)
Supplement: S3 Table — (DOCX) [file pone.0244066.s003.docx]

**S3 Table. Comparison of sociodemographic characteristics by viral load category and gender.**

|  | Male | | Female | | p-value (males) | p-value (females) |  |
| --- | --- | --- | --- | --- | --- | --- | --- |
|  | **Cases (72)** | **Controls (47)** | **Cases (83)** | **Controls (105)** |  |  |  |
| Civil status |  |  |  |  | **0.021** | 0.324 |  |
| Married | 26 (36.1) | 29 (61.7) | 26 (31.3) | 41 (39.0) | Ref | Ref |  |
| Single | 24 (33.3) | 9 (19.1) | 11 (13.3) | 8 (7.6) | **0.022** | 0.143 |  |
| Divorced | 17 (23.6) | 4 (8.5) | 27 (32.5) | 27 (25.7) | **0.012** | 0.218 |  |
| Widowed | 5 (6.9) | 5 (10.6) | 19 (22.9) | 29 (27.6) | 0.874 | 0.933 |  |
| Ethnicity |  |  |  |  | 0.588 | 0.309 |  |
| Oromo | 39 (54.2) | 29 (61.7) | 39 (47.0) | 38 (36.2) | Ref | Ref |  |
| Amhara | 17 (23.6) | 11 (23.4) | 31 (37.3) | 45 (42.9) | 0.870 | 0.222 |  |
| Other | 16 (22.2) | 7 (14.9) | 13 (15.7) | 22 (21.0) | 0.588 | 0.186 |  |
| Number of languages spoken (median, IQR) | 2 (1-2) | 2 (1-3) | 2 (1-2) | 2 (1-2) | 0.932 | 0.572 |  |
| Religion |  |  |  |  | 0.187 | 0.923 |  |
| Orthodox | 56 (77.8) | 32 (68.1) | 57 (68.7) | 74 (70.5) | Ref | Ref |  |
| Protestant | 9 (12.5) | 12 (25.5) | 19 (22.9) | 21 (20.0) | 0.086 | 0.657 |  |
| Muslim | 7 (9.7) | 3 (6.4) | 6 (7.2) | 10 (9.5) | 0.691 | 0.647 |  |
| Other | 0 | 0 | 1 (1.2) | 0 | - | - |  |
| Education |  |  |  |  | 0.372 | 0.753 |  |
| No education | 11 (15.3) | 6 (12.8) | 20 (24.1) | 28 (26.7) |  |  |  |
| Primary (1-8) | 34 (47.2) | 27 (57.4) | 37 (44.6) | 39 (37.1) |  |  |  |
| Secondary (9-12) | 18 (25.0) | 6 (12.8) | 21 (25.3) | 32 (30.5) |  |  |  |
| Tertiary^†^ | 9 (12.5) | 8 (17.0) | 5 (6.0) | 6 (5.7) |  |  |  |
| Employment status |  |  |  |  | 0.115 | **0.023** |  |
| Formal employment | 25 (34.7) | 23 (48.9) | 11 (13.3) | 27 (25.7) | Ref | Ref |  |
| Self-employed | 13 (18.1) | 11 (23.4) | 22 (26.5) | 20 (19.0) | 0.867 | 0.036 |  |
| Daily labor | 19 (26.4) | 6 (12.8) | 21 (25.3) | 15 (14.3) | 0.052 | **0.012** |  |
| Housewife | 0 | 0 | 14 (16.9) | 28 (26.7) | - | 0.673 |  |
| Student | 9 (12.5) | 1 (2.1) | 8 (9.6) | 3 (2.9) | 0.053 | **0.014** |  |
| Unemployed^‡^ | 6 (8.3) | 6 (12.8) | 7 (8.4) | 12 (11.4) | 0.897 | 0.546 |  |
| Job security |  |  |  |  | 0.080 | 0.183 |  |
| Seldom or never concerned | 59 (81.9) | 44 (93.6) | 64 (77.1) | 89 (84.8) |  |  |  |
| Regularly concerned | 13 (18.1) | 3 (6.4) | 19 (22.9) | 16 (15.2) |  |  |  |
| Work type^§^ |  |  |  |  | 0.110 | 0.361 |  |
| No work | 9 (12.5) | 1 (2.1) | 22 (26.5) | 30 (28.6) | Ref | Ref |  |
| Agriculture | 6 (8.3) | 5 (10.6) | 4 (4.8) | 3 (2.9) | 0.097 | 0.463 |  |
| Transportation | 13 (18.1) | 4 (8.5) | 0 | 0 | 0.396 | - |  |
| Retail/petty trade | 6 (8.3) | 7 (14.9) | 33 (39.8) | 27 (25.7) | **0.049** | 0.181 |  |
| Public office/education | 3 (4.2) | 6 (12.8) | 0 | 3 (2.9) | **0.023** | - |  |
| Security/military | 9 (12.5) | 8 (17.0) | 0 | 0 | 0.073 | - |  |
| Construction | 20 (27.8) | 7 (14.9) | 4 (4.8) | 2 (1.9) | 0.315 | 0.270 |  |
| Housework/cleaning | 0 | 0 | 14 (16.9) | 24 (22.9) |  | 0.601 |  |
| Industry/skilled labor | 4 (5.6) | 6 (12.8) | 2 (2.4) | 5 (4.8) | **0.035** | 0.325 |  |
| Other professions^¶^ | 2 (2.8) | 5 (4.2) | 4 (4.8) | 11 (10.5) | 0.062 | 0.566 |  |
| Job Location |  |  |  |  | 0.341 | 0.280 |  |
| Within residential district | 48 (66.7) | 37 (78.7) | 75 (90.4) | 101 (96.2) | Ref | Ref |  |
| Single location outside residential district | 10 (13.9) | 5 (10.6) | 5 (6.0) | 3 (2.9) | 0.463 | 0.446 |  |
| Multiple locations outside residential district | 14 (19.4) | 5 (10.6) | 3 (3.6) | 1 (1.0) | 0.173 | 0.248 |  |
| *Household* |  |  |  |  |  |  |  |
| Job commute |  |  |  |  | 0.199 | 0.445 |  |
| Home every day | 56 (77.8) | 41 (87.2) | 81 (97.6) | 104 (99.0) |  |  |  |
| Home less often | 16 (22.2) | 6 (12.8) | 2 (2.4) | 1 (1.0) |  |  |  |
| Monthly household income |  |  |  |  | **0.040** | **0.004** |  |
| <35 USD | 40 (55.6) | 17 (36.2) | 60 (72.3) | 54 (51.4) |  |  |  |
| ≥35 USD | 32 (44.4) | 30 (63.8) | 23 (27.7) | 51 (48.6) |  |  |  |
| Residence ownership |  |  |  |  | **0.016** | 0.387 |  |
| Owned | 18 (25.0) | 22 (46.8) | 25 (30.1) | 24 (22.9) | Ref | Ref |  |
| Rented | 37 (51.4) | 22 (46.8) | 44 (53.0) | 66 (62.9) | 0.084 | 0.197 |  |
| Owned by family/others | 17 (23.6) | 3 (6.4) | 14 (16.3) | 15 (14.3) | **0.006** | 0.815 |  |
| Number of rooms (median, IQR) | 2 (1-3) | 2 (1-3) | 2 (1-3) | 2 (1-2) | 0.489 | 0.375 |  |
| Household members (median, IQR) | 3 (1-5) | 3 (2-5) | 3 (2-4) | 3 (2-4) | 0.735 | 0.109 |  |
| Number of children (median, IQR) | 1 (0-2) | 2 (2-4) | 2 (1-3) | 1 (1-2.5) | **0.005** | 0.289 |  |
| Residence in rural area |  |  |  |  |  |  |  |
| Yes | 2 (2.8) | 1 (2.1) | 7 (8.4) | 6 (5.7) | 0.825 | 0.468 |  |
| No | 70 (97.2) | 46 (97.9) | 76 (91.6) | 99 (94.3) |  |  |  |
| Urban wealth quintile^††^ |  |  |  |  | 0.115 | 0.058 |  |
| 1st (poorest) | 6 (9.1) | 2 (4.7) | 10 (12.5) | 5 (4.9) | Ref | Ref |  |
| 2^nd^ | 14 (21.2) | 7 (16.3) | 19 (23.8) | 18 (17.6) | 0.666 | 0.317 |  |
| 3^rd^ | 27 (40.9) | 11 (25.6) | 27 (33.8) | 27 (26.5) | 0.822 | 0.257 |  |
| 4^th^ | 8 (12.1) | 13 (30.2) | 11 (13.8) | 24 (23.5) | 0.089 | **0.025** |  |
| 5th (richest) | 11 (16.7) | 10 (23.3) | 13 (16.3) | 28 (27.5) | 0.279 | **0.023** |  |
| Lower three urban quintiles | 47 (71.2) | 20 (46.5) | 56 (70.0) | 51 (50.0) | **0.011** | **0.007** |  |

Binominal logistic regression for categorical variables. Mann-Whitney U test for continuous variables. Data reported in absolute number and percentage (in brackets) and unadjusted odds ratios, unless otherwise specified. IQR: Interquartile range; USD: United States dollar.

† Including Technical and Vocational Education and Training (TVET) and university degrees

‡ Including unpaid volunteers work and pension/retirement

§ Including previous work for people currently unemployed

¶ Commercial sex work (n=2), finance (n=3), healthcare (n=3), server (n=5), (assistant) chef (n=4), cosmetics and haircare (n=2)

†† Sub analysis on urban study population: cases (n=146) and controls (n=145).
