# Supplementary material for: Socio-economic condition and lack of virological suppression among adults and adolescents receiving antiretroviral therapy in Ethiopia
Source: PLoS One. 2020 Dec 15;15(12):e0244066. doi: 10.1371/journal.pone.0244066 (PMC7737988; doi:10.1371/journal.pone.0244066)
Supplement: S4 Table — (DOCX) [file pone.0244066.s004.docx]

**S4 Table.** **Comparison of physical, mental and sexual health, including risky sexual behavior and substance use by viral load category and gender**

|  | Male | | Female | | p-value (males) | p-value (females) | |  |
| --- | --- | --- | --- | --- | --- | --- | --- | --- |
|  | **Cases (72)** | **Controls (47)** | **Cases (83)** | **Controls (105)** |  |  |  |  |
| Self-rated wellbeing |  |  |  |  | 0.327 | **0.001** |  |  |
| Excellent | 53 (73.6) | 43 (91.5) | 49 (59.0) | 88 (83.8) | Ref | Ref |  |  |
| Suboptimal | 15 (20.8) | 4 (8.5) | 16 (19.3) | 8 (7.6) | 0.063 | **0.006** |  |  |
| Poor | 4 (5.6) | 0 | 18 (21.7) | 9 (8.6) | **-** | **0.004** |  |  |
| Self-reported illness last month |  |  |  |  | 0.824 | **0.005** |  |  |
| 0-3 days | 65 (90.3) | 43 (91.5) | 63 (75.9) | 96 (91.4) |  |  |  |  |
| ≥4 days | 7 (9.7) | 4 (8.5) | 20 (24.1) | 9 (8.6) |  |  |  |  |
| Depressive symptoms (PHQ-2 score) |  |  |  |  | 0.973 | 0.208 |  |  |
| No (0) | 66 (91.7) | 43 (91.5) | 71 (85.5) | 96 (91.4) |  |  |  |  |
| Yes (≥1) | 6 (8.3) | 4 (8.5) | 12 (14.5) | 9 (8.6) |  |  |  |  |
| Major depressive disorder (n=5 [PHQ-2 score ≥3]; PHQ-9 score ≥8) | 1 (1.4) | 0 | 1 (1.2) | 1 (1.0) |  |  |  |  |
| *Sexual health* |  |  |  |  |  |  |  |  |
| Number of lifetime sexual partners |  |  |  |  | **0.004** | **<0.001** |  |  |
| 1-5 | 45 (62.5) | 22 (46.8) | 46 (55.4) | 89 (84.8) | Ref | Ref |  |  |
| 6-10 | 3 (4.2) | 2 (4.3) | 9 (10.8) | 5 (4.8) | 0.744 | **0.033** |  |  |
| >10 | 11 (15.3) | 21 (44.7) | 20 (24.1) | 6 (5.7) | **0.003** | **<0.001** |  |  |
| Never sexually active | 13 (18.1) | 2 (4.3) | 8 (9.6) | 5 (4.8) | 0.150 | 0.059 |  |  |
| Age at sexual debut (median, IQR) | 18 (15-20) | 20 (17.5-21.5) | 18 (16-20) | 17 (15-20) | **0.040** | 0.104 |  |  |
| History of STI^†^ |  |  |  |  | **<0.001** | 0.060 |  |  |
| Yes | 16 (22.2) | 27 (57.4) | 18 (21.7) | 12 (11.4) |  |  |  |  |
| No | 56 (77.8) | 20 (42.6) | 65 (78.3) | 93 (88.6) |  |  |  |  |
| Sexually active,  last month |  |  |  |  | **0.008** | 0.485 |  |  |
| Yes | 9 (15.5) | 18 (39.1) | 18 (24.0) | 29 (28.7) |  |  |  |  |
| No | 49 (84.5) | 28 (60.9) | 57 (76.0) | 72 (71.3) |  |  |  |  |
| *Substance use* |  |  |  |  |  |  |  |  |
| Alcohol consumption |  |  |  |  | 0.490 | 0.579 |  |  |
| Less than once a year | 41 (56.9) | 27 (57.4) | 59 (71.1) | 78 (74.3) | Ref | Ref |  |  |
| Once a month or less | 14 (19.4) | 11 (23.4) | 20 (24.1) | 21 (20.0) | 0.709 | 0.519 |  |  |
| 2-4 times a month | 8 (11.1) | 7 (14.9) | 3 (3.6) | 2 (1.9) | 0.620 | 0.461 |  |  |
| 2 or more times a week | 9 (12.5) | 2 (4.3) | 1 (1.2) | 4 (3.8) | 0.185 | 0.328 |  |  |
| Hazardous drinking  (FAST Score) |  |  |  |  | **0.042** | 0.178 |  |  |
| Yes (≥3) | 17 (23.6) | 4 (8.5) | 6 (7.2) | 3 (2.9) |  |  |  |  |
| No (<3) | 55 (76.4) | 43 (91.5) | 77 (92.8) | 102 (97.1) |  |  |  |  |
| Harmful alcohol use (n=29 [FAST score ≥3]; AUDIT score ≥8) | 8 (11.1) | 2 (4.3) | 1 (1.2) | 1 (1.0) | 0.204 | 0.867 |  |  |
| Regular *Khat* use^‡^ |  |  |  |  | 0.210 | 0.178 |  |  |
| Yes | 12 (16.7) | 4 (8.5) | 6 (7.2) | 3 (2.9) |  |  |  |  |
| No | 60 (83.3) | 43 (91.5) | 77 (92.8) | 102 (97.1) |  |  |  |  |

Binominal logistic regression for categorical variables. Mann-Whitney U test for continuous variables. Data reported in absolute number and percentage (in brackets) and unadjusted odds ratios, unless otherwise specified. IQR: Interquartile range; PHQ: Patient Health Questionnaire; STI: Sexually Transmitted Infection; FAST: Fast Alcohol Screening Test; AUDIT: Alcohol Use Disorder Identification Test.

† History of symptoms of gonorrhea-like discharge, chancre-like lesion or genital itchiness

‡ Amphetamine-like plant substance ingested through chewing
