# Supplementary material for: Socio-economic condition and lack of virological suppression among adults and adolescents receiving antiretroviral therapy in Ethiopia
Source: PLoS One. 2020 Dec 15;15(12):e0244066. doi: 10.1371/journal.pone.0244066 (PMC7737988; doi:10.1371/journal.pone.0244066)
Supplement: S5 Table — (DOCX) [file pone.0244066.s005.docx]

**Multivariable regression model**

1. Unmatched univariate analysis was performed on continuous (Mann-Whitney U test) and categorical variables (logistic regression). For univariate analysis. variables with p<0.25 were added to a multivariable logistic regression model.
2. Variables showing multicollinearity (variance inflation factor [VIF]>5 in linear logistic regression including independent dummy categorical and continuous variables) or for which the proportion of missing data was >10%, as well as those that only concerned a minor subgroup of participants were excluded; this corresponded to the following variables: work type, recent CD4 count, AUDIT score and sexual behaviour variables within the last month.
3. The model was adapted through a stepwise backwards elimination protocol as cited in the methods section (table 1). Variables with p<0.10 were kept in the model. as well as variables for which elimination led to >15% confounding effect on remaining variables.
4. All eliminated variables were then re-entered, and those with p<0.15 were kept in the model. The final model is presented in the manuscript.

**S5 Table. Steps in backwards elimination protocol for multivariable regression model.**

| Step | | | Regression coefficient of constant | Removed variable | | p-value removed variable | Goodness of fit (H&L) | Change in coefficient of constant | | | Comment |  |
| --- | --- | --- | --- | --- | --- | --- | --- | --- | --- | --- | --- | --- |
| 1 | -1.872 | | | ART clinic location | 0.998 | | 0.636 |  | | | Removed |  |
| 2 | | | -1.873 | | Child number | 0.830 | | 0.636 | | 1.000534 | Removed | |
| 3 | | | -1.884 | | Civil status | 0.725 | | 0.804 | | 1.005873 | Removed | |
| 4 | | | -1.634 | | Employment status | 0.463 | | 0.699 | | 0.867304 | Removed | |
| 5 | | | -1.234 | | Transfer in | 0.418 | | 0.861 | | **0.755202** | **Employment status kept in equation** | |
| 6 | | | -1.565 | | Job concern | 0.427 | | 0.561 | | 0.957772 | Removed | |
| 7 | | | -1.440 | | Residence status | 0.342 | | 0.643 | | 0.920128 | Removed | |
| 8 | | | -1.859 | | illness | 0.299 | | 0.193 | | **1.290972** | **Residence status kept in equation** | |
| 9 | | | -1.475 | | Job commute | 0.254 | | 0.826 | | 1.024306 | Removed | |
| 10 | | | -1.489 | | Khat | 0.153 | | 0.793 | | 1.009492 | Removed | |
| 11 | | | -1.415 | | ART regimen | 0.139 | | 0.577 | | 0.950302 | Removed | |
|  | | |  | | **Removed variable** | **p-value added variable** | |  | |  |  | |
| 12 | | | -1.503 | | Child number | 0.693 | |  | | 0.964664 | Not added | |
| 12 | | |  | | ART same woreda | 0.952 | |  | |  | Not added | |
| 13 | | |  | | Partner status | 0.732 | |  | |  | Not added | |
| 14 | | |  | | Transfer in | 0.520 | |  | |  | Not added | |
| 15 | | |  | | Job concern | 0.457 | |  | |  | Not added | |
| 16 | | |  | | Illness | 0.287 | |  | |  | Not added | |
| 17 | | |  | | Travel back home | 0.234 | |  | |  | Not added | |
| 18 | | |  | | Khat | 0.172 | |  | |  | Not added | |
| 19 | | |  | | ART regimen | 0.139 | |  | |  | **ART regimen kept in equation** | |

ART: Antiretroviral therapy; AUDIT: Alcohol Use Disorder Identification Test; H&L: Hosmer-Lemeshow test.
