# Supplementary material for: Socio-economic condition and lack of virological suppression among adults and adolescents receiving antiretroviral therapy in Ethiopia
Source: PLoS One. 2020 Dec 15;15(12):e0244066. doi: 10.1371/journal.pone.0244066 (PMC7737988; doi:10.1371/journal.pone.0244066)
Supplement: Attachment — Submitted filename: Martin Plymoth rebuttal letter1.docx [file pone.0244066.s006.docx]

PONE-D-20-07483
Socio-economic condition and lack of virological suppression among adults and adolescents receiving antiretroviral therapy in Ethiopia

**Response letter**

Dear Dr. Jianhong Zhou,

We appreciate your review of our manuscript and the comments provided by the two external reviewers. Below, we have addressed these comments point by point. For most comments, we have followed the advice given by the reviewers. However, for certain comments we prefer to keep the original formulations in the submitted manuscript, and we have explained these reasons in the replies inserted below.

We hope that our revision of the manuscript will be to your satisfaction, as well as the replies provided, and that the manuscript will meet criteria for publication in PLOS One.

Yours sincerely,

Martin Plymoth and Per Björkman

 Reviewer #1:

**Reviewer’s comment**

I read with interest this manuscript.
I think that is well wrote and with a good idea research from an important setting (Ethiopia)
Only some suggestions:

1. Introduction: line 44 is the definition of social determinants of health (cite Marmot et al on this issue)

***Authors’ response***

*We appreciate this suggestion, and have modified this part accordingly.*

**Reviewer’s comment**

join line 55-56; delete line 71-72

***Authors’ response***

*We have revised the manuscript accordingly.*

**Reviewer’s comment**

2. Methods: write the time of the study.

***Authors’ response***

*The study was conducted October-December 2018, as indicated in the methods section (line 110). Participants were considered for inclusion if they had available viral load data after starting ART. Universal viral load testing was gradually introduced at the study clinics since 2015. For the purpose of this study, we did not use a pre-defined time period for patient registration in the study clinics.*

**Reviewer’s comment**

3. Results:well wrote. Delete line189

***Authors’ response***

*Due to the current font and formal of the manuscript, we have not been able to correct this in the word document. We hope that this can be corrected at time of print.*

**Reviewer’s comment**
4. Discussion: well wrote. Compare with other study of low setting (ec cite Prevalence and Predictors of Malaria in Human Immunodeficiency Virus Infected Patients in Beira, Mozambique. Int J Environ Res Public Health. 2018;15(9):2032. Published 2018 Sep 17 and The At Risk Child Clinic (ARCC): 3 Years of Health Activities in Support of the Most Vulnerable Children in Beira, Mozambique. Int J Environ Res Public Health. 2018;15(7):1350. Published 2018 Jun 27. doi:10.3390/ijerph15071350) who underline how women and child are the most vulenrable group and the comorbidity and co-infection with Malaria can be devasting in people with HIV.

Furhermore, as for HIV also TB responde to social determinats of health. (doi:10.1016/j.tube.2017.01.002 and doi:10.1186/s13104-018-3209-9)

***Authors’ response***

*We have revised the discussion according to these suggestions, and have included references with data on associations between socio-economic conditions and outcomes for other communicable diseases (TB and malaria, as suggested). We acknowledge the association between socio-economic condition and malaria prevalence shown in the suggested references, but since these articles do not report associations with treatment outcome, we have chosen not to include these here. Instead, we have included a reference to a recently published systematic review and meta-analysis on interactions between socio-economic status and malaria burden in sub-Saharan Africa ([1, 2]; ref. 42 and ref. 45 in manuscript).*

**Reviewer’s comment**

5. Conclusion: improve with a advocacy that only pharmacological approach is not sufficient to control burden of HIV

***Authors’ response***

*We appreciate this comment, and have revised the conclusion accordingly.*

**Reviewer’s comment**

6. Table and statistical analysis are very well done. Congratulations

Reviewer #2:

**Reviewer’s comment**

Introduction
*“Since such factors might be associated with risk of HIV transmission, we also investigated whether lack of virological suppression during ART is associated with high-risk sexual behaviour.”*
Yes, socioeconomic factors might be associated with HIV transmission, but I don’t see why you use that fact to support your secondary aim, you cannot use that fact to motivate your reason for investigating the association between virological suppression during ART and high-risk behaviour (you may look for a reference that supports this association), rather simply state that: A secondary aim of our study was to investigate the association between virological suppression and high-risk sexual behaviour.

***Authors’ response***

*Socio-economic factors have previously been reported to influence HIV transmission in different settings. In the formulation of our aims, we explained the motivation for exploring the relationship between high-risk behaviour and virological suppression ( … Since such factors might be associated with risk of HIV transmission, we also investigated whether lack of virological suppression during ART is associated with high-risk sexual behavior ...). We acknowledge that this objective was secondary to the first aim stated (association between virological suppression and socio-economic factors). We have considered the suggestion put forth, but we think that the current way of formulation is preferable to that suggested by the reviewer, and that it will make it easier for readers to understand why we included this research question in our study. For this reason, we would prefer to keep the original text.*

**Reviewer’s comment**

*“For this purpose, we performed a case-control study of ART recipients at Ethiopian public health facilities, comparing persons with VL>1000 copies/ml to those with virological suppression.”*
To me, that statement makes it as if you are conducting a solely comparative study, it completely drifts away from your title and the introduction. Nowhere in the introduction do you talk about virologically suppressed Vs non-suppressed. Besides the fact that you are focusing attention on the association between lack of virological suppression and socioeconomic condition, the comparative factor strengthens your study even further. If you are comparing, this aspect of your study must “come out” in the study title, introduction and the aims. WHY ARE YOU COMPARING THE VIROLOGICALLY SUPPRESSED VS. NON SUPPRESSED? To have a referral point? Then say so. WOULD YOU HAVE FOUND DIFFERENT RESULTS IF YOU HAD NOT COMPARED, i.e., if you had only sought for associations between socioeconomic conditions and those with VL>1000 copies/ml?
Alternatively, your sentence could read: In order to understand the socioeconomic conditions impacting on virological suppression, we performed a case-control study of ART recipients at Ethiopian public health facilities, comparing persons with VL>1000 copies/ml to those with virological suppression.

***Authors’ response***

*As explained above, our primary aim was to investigate the association between socio-economic conditions and virological suppression. Different study designs can be considered for this purpose. Our research group has previously studied factors associated with virological suppression in adults starting ART recruited from a prospective cohort [3, 4]. Although a cohort study design has certain benefits, it may be less representative of the true population of HIV-positive patients receiving care (due to selection bias and loss to follow-up, for example). For the current study, we therefore chose to use a case-control design. For this purpose, it is necessary to define both case and control groups. We consider persons with viral load data indicating persistent virological suppression to be most suitable as a control group for studying the association between lack of virological suppression and the independent variable of interest (socio-economic conditions).*

*Since reviewer 1 recommended deletion of this sentence in the introduction, we chose to follow this advice.*

**Reviewer’s comment**
Methods, results and ethical considerations
Reviewer comments
Comprehensive and well-articulated.

Discussion
The discussion is adequate. However, you fail to compare your findings with other studies that have also ‘compared’ virologically suppressed vs. non-suppressed.

***Authors’ response***

*We appreciate this suggestion, and have revised the discussion accordingly, with addition of further references to studies that compare virologically suppressed and non-suppressed ART recipients in sub-Saharan Africa ([5, 6]; ref. 67 and ref. 68). However, as pointed out previously, previous data on association between virological suppression and socio-economic condition from this world region is limited.*

**Reviewer’s comment**

General comments
All in all, this is a good study, and I praise you for the good work.

**References**

1.	Duarte R, Lönnroth K, Carvalho C, Lima F, Carvalho ACC, Muñoz-Torrico M, et al. Tuberculosis, social determinants and co-morbidities (including HIV). Pulmonology. 2018;24(2):115-9.

2.	Degarege A, Fennie K, Degarege D, Chennupati S, Madhivanan P. Improving socioeconomic status may reduce the burden of malaria in sub Saharan Africa: A systematic review and meta-analysis. PLoS One. 2019;14(1):e0211205.

3.	Reepalu A, Balcha TT, Sturegard E, Medstrand P, Bjorkman P. Long-term Outcome of Antiretroviral Treatment in Patients With and Without Concomitant Tuberculosis Receiving Health Center-Based Care-Results From a Prospective Cohort Study. Open forum infectious diseases. 2017;4(4):ofx219.

4.	Reepalu A, Balcha TT, Skogmar S, Isberg PE, Medstrand P, Bjorkman P. Development of an algorithm for determination of the likelihood of virological failure in HIV-positive adults receiving antiretroviral therapy in decentralized care. Global health action. 2017;10(1):1371961.

5.	Desta AA, Woldearegay TW, Futwi N, Gebrehiwot GT, Gebru GG, Berhe AA, et al. HIV virological non-suppression and factors associated with non-suppression among adolescents and adults on antiretroviral therapy in northern Ethiopia: a retrospective study. BMC Infect Dis. 2020;20(1):4.

6.	Bulage L, Ssewanyana I, Nankabirwa V, Nsubuga F, Kihembo C, Pande G, et al. Factors Associated with Virological Non-suppression among HIV-Positive Patients on Antiretroviral Therapy in Uganda, August 2014-July 2015. BMC Infect Dis. 2017;17(1):326.
