# Supplementary material for: Socio-economic condition and lack of virological suppression among adults and adolescents receiving antiretroviral therapy in Ethiopia
Source: PLoS One. 2020 Dec 15;15(12):e0244066. doi: 10.1371/journal.pone.0244066 (PMC7737988; doi:10.1371/journal.pone.0244066)
Supplement: Attachment — Submitted filename: Martin Plymoth rebuttal letter_201120.docx [file pone.0244066.s007.docx]

PONE-D-20-07483R1
Socio-economic condition and lack of virological suppression among adults and adolescents receiving antiretroviral therapy in Ethiopia

**Response letter**

Dear Dr. Kennedy Otwombe,

We appreciate your review of our manuscript and the comments provided by the two external reviewers. Below, we have addressed these comments point by point.

We hope that our revision of the manuscript will be to your satisfaction, as well as the replies provided, and that the manuscript will meet criteria for publication in PLOS One.

Yours sincerely,

Martin Plymoth and Per Björkman

 Reviewer #3:

3. Has the statistical analysis been performed appropriately and rigorously?

**Reviewer’s comment**

I don’t know.

***Authors’ response***

*Since the reviewer does not elaborate further, we assume this to be in relation to the Holm-Sidak correction for multiple comparisons, which we address in detail below.*

**Reviewer’s comment**

Pages 15-18 mentions several significant p-values, yet no mention applying Holm-Sidak. Exactly how were these multiple p-values addressed to minimize Type 1 error?

Table 4 p value are those post-Holm Sidak? Better to see the original p-values apply the Holm-Sidak, then show the outcome.

Other tables show the p-values as well, we they not included in the Holm-Sidak correction?

The Holm-Sidak application confuses. Please clarify…

***Authors’ response***

*Page 15-18 (Results section, manuscript page 8-11) presents the results from our univariate analysis of collected data (including a gender-disaggregated analysis), as well as the results from our multivariable analysis model.*

*The primary objective of our unmatched univariate analysis was to find variables with a p-value <0.25, which could potentially be significant in a multivariable analysis. Correction for multiple comparisons to correct for type I error was not performed in our univariate analysis, instead this was applied to the multivariable model. This decision was based on the protocol previously cited in our Method section, i.e. a purposeful selection of covariates [1]. All significant results commented on in our discussion derives from our multivariable analysis, which has been corrected for multiple comparisons using the Holm-Sidak Method.*

*The Holm-Sidak Method is a method to adjust for multiple comparisons (reduce type I error) and is similar to the more well-known Bonferroni correction [2].*

*In table 4, original p-values are displayed in the column “multivariable p-values”, significant p-values after Holm-Sidak correction (as annotated in the footnote) are displayed in the column “Adjusted p-value”. Other tables show results from univariate analysis, no correction for multiple comparisons has been made.*

**Reviewer’s comment**

There are still problems with the abbreviations, where a number of them were given at first mention without presenting the meaning.

***Authors’ response***

*We have gone through the text and written out the following abbreviation: USD, United States Dollar. The following abbreviations have not been written out due to their common nature and in accordance with previous published works in PLOS ONE: HIV, RNA, CD4.*

1.	Bursac Z, Gauss CH, Williams DK, Hosmer DW. Purposeful selection of variables in logistic regression. Source code for biology and medicine. 2008;3:17.

2.	GraphPad. How the Holm-Sidak method works [cited 21-11-2020. Available from: <https://www.graphpad.com/guides/prism/7/statistics/stat_how_the_holm_method_woks.htm>].
